# Differentiation between suicide attempt and suicidal ideation in patients with major depressive disorder using cortical functional network

**DOI:** 10.1192/j.eurpsy.2025.528

**Published:** 2025-08-26

**Authors:** S. Shim

**Affiliations:** psychiatry, soonchunhyang university cheonan hospital, cheonan, Korea, Republic Of

## Abstract

**Introduction:**

The detection of electrophysiological markers to differentiate patients with suicide attempts could support the efforts of clinicians to provide proper diagnosis and treatment for them, and finally reduce the suicide mortality rate.

**Objectives:**

The objective of the current study was to investigate and compare cortical functional networks from resting-state electroencephalogram in patients with suicide attempts and suicidal ideation using source-level weighted network analysis. We also examined the possibility of network features serving as biomarkers by differentiation between suicide attempts and suicidal ideation using machine learning techniques.

**Methods:**

This study investigated source-level cortical functional networks using resting-state electroencephalography in drug-naïve depressed patients with suicide attempt and suicidal ideation. Electroencephalogram was recorded in 55 patients with suicide attempts and in 54 patients with suicidal ideation. Graph-theory-based source-level weighted functional networks were assessed using strength, clustering coefficient (CC), and path length (PL) in seven frequency bands. Finally, we applied machine learning to differentiate between the two groups using source-level network features.

**Results:**

There were significant differences in the three global level indices of the high alpha band. The strength and CC of the high alpha band were significantly lower in patients with suicide attempts than in those with suicide attempts ideation. In contrast, the PL of the high alpha band was significantly higher in patients with suicide attempts than in those with suicide ideation. No significant differences in the other frequency bands were between the two groups.

The nodal CCs of patients with suicide attempts were significantly lower than those of patients with suicide ideation in most regions, except for three of the 68 regions.

The best classification performance between suicide attempts and suicide ideation was an accuracy of 73.39%, a sensitivity of 76.36%, and a specificity of 70.37% with 17 features. Three of these features were global-level network indices (strength, CC, and PL) in the high alpha band; the other 14 features were high alpha band nodal level CCs in limbic, frontal, temporal, parietal,and occipital area.

**Image 1:**

**Figure:**
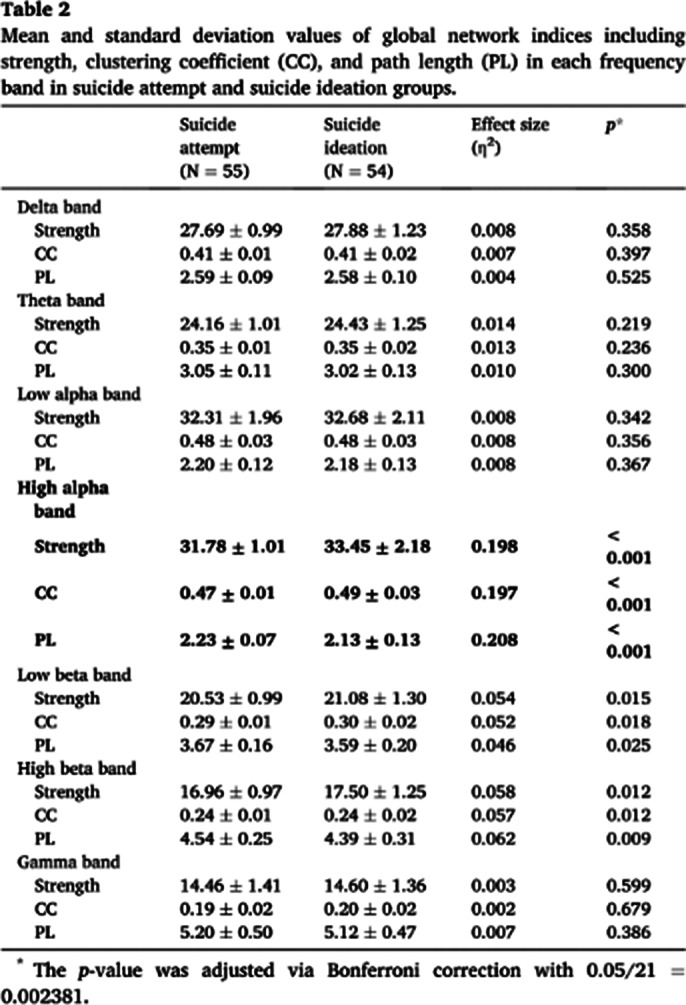

**Image 2:**

**Figure:**
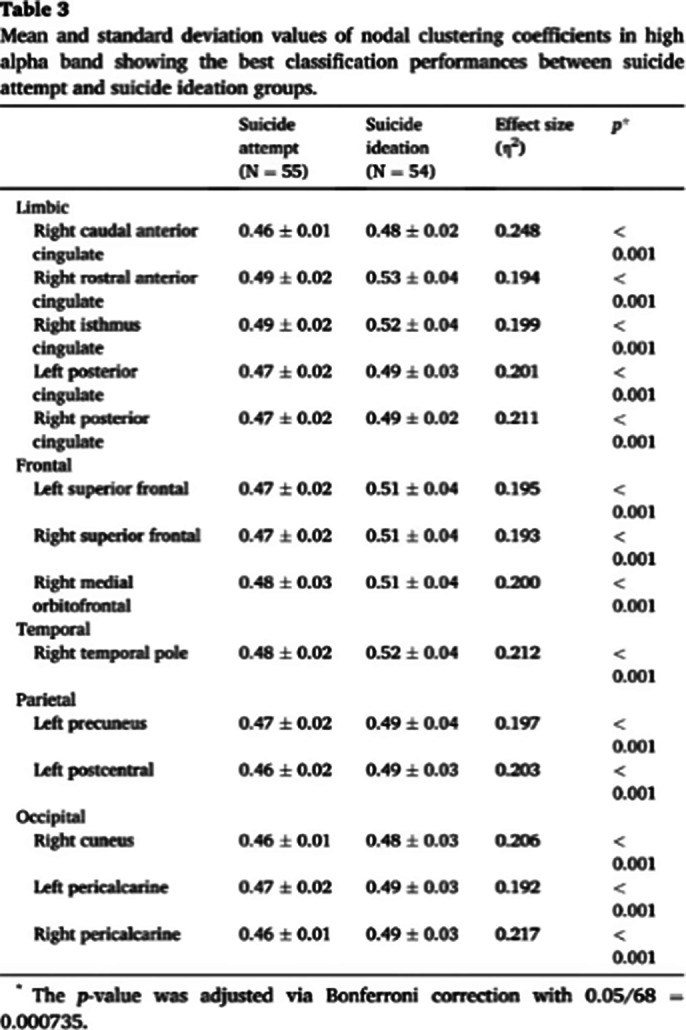

**Image 3:**

**Figure:**
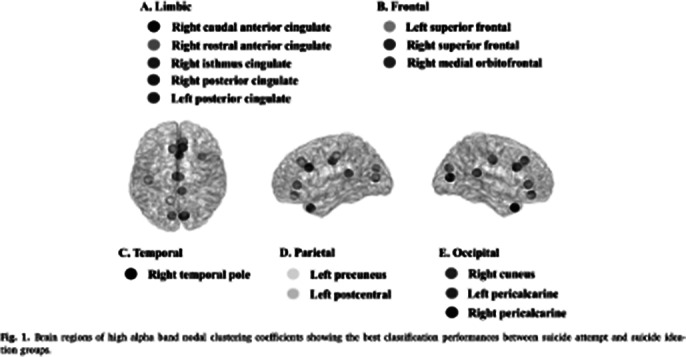

**Conclusions:**

This is the first study to use electroencephalogram source level network measures, revealing that network indices for high alpha band could be potential biomarkers to distinguish betweensuicide attempts and suicide ideation in patients with depression. Moreover, our study evaluated the electroencephalogram signals immediately after fatal suicide attempts in a relatively large number of un-medicated patients.

**Disclosure of Interest:**

None Declared

